# Entrepreneurial Curiosity, Innovativeness of the Entrepreneur, and Company Growth

**DOI:** 10.3390/bs12110424

**Published:** 2022-10-31

**Authors:** Žiga Peljko, Jasna Auer Antončič

**Affiliations:** 1 KD Group d.d., SI-1000 Ljubljana, Slovenia; 2 Faculty of Management, University of Primorska, SI-6000 Koper, Slovenia

**Keywords:** entrepreneurial curiosity, innovativeness, entrepreneur, growth, entrepreneurship, SMEs

## Abstract

Researchers have studied entrepreneurial curiosity and innovativeness as determinants of entrepreneurial behavior but have not linked them with company growth in a model. The intention of this enquiry was to examine the associations between the entrepreneur’s psychological constructs of entrepreneurial curiosity and innovativeness and business growth, as examined by the conceptualization and analysis of hypotheses. For this research, data were obtained via a survey questionnaire. The sample consisted of 851 entrepreneurs of companies in three European countries. Each company had 250 workers or fewer. This study contributes to the entrepreneurship knowledge base by presenting empirical testimony on the associations between entrepreneurial curiosity, entrepreneurial innovativeness, and firm growth, as well as presenting advanced cross-nationally analogous measurement instruments of entrepreneurial curiosity and innovativeness. The entrepreneur’s curiosity is important for their innovativeness, and this innovativeness is essential for business growth.

## 1. Introduction

Entrepreneurship involves creating new enterprises and ventures and improving existing ones, signifies an essential part in both advanced and emerging countries, and contributes to economic dynamism and progress [1,2]. The occurrence of entrepreneurship is deep-rooted in expressions of business importance [3]. Research [4,5,6] confirms that entrepreneurship creates new jobs, reducing unemployment and fostering economic development [7,8,9]. The entrepreneurship framework allows the study of how growth is manifested as an element of a company’s success [10].

An important question for entrepreneurs is why some enjoy success, but others do not. In this direction, entrepreneurship studies mostly center on entrepreneurial success and the entrepreneur’s behavioral patterns or personality traits [11]. Some research also examines entrepreneurs’ performance in terms of the performance of their company and whether the entrepreneur can help generate business [12].

Coad and Hölzl noted the interest of both individual companies (interested in sales growth) and policymakers (interested in creating new jobs) in studying the determinants that effect company growth [13]. While sales growth is the most influenced by short- and long-term changes in the company, with these perhaps being the most common indicators that managers and entrepreneurs rely on to measure growth, employment also brings some advantages as an indicator of company growth [13]. Accordingly, Auer Antončič and Antončič stated that company growth is an important factor of business success and is based on employee efficiency [14].

Researchers have studied entrepreneurial curiosity [15,16,17,18] and innovativeness [19,20,21] as determinants influencing entrepreneurial behavior but have, to date, not linked them with company growth in a model. The goal of this investigation was to examine the associations between the entrepreneur’s psychological constructs of entrepreneurial curiosity and innovativeness and business growth. The principal objective was to fill the gaps in the literature in terms of the association between the curiosity and innovativeness of the entrepreneur and the company’s growth.

As per our review of the scientific and professional literature, how entrepreneurial curiosity affects entrepreneurial innovation and how it affects company growth remain to be explored. Enterprise growth is the soundest indicator of entrepreneurial success and performance (refer to [22,23,24,25] for examples). Determinants of entrepreneurial success are hard to identify [26]. Some recent studies have covered entrepreneurial curiosity in relationship to cognition and uncertainty [27]; innovativeness, passion, and entrepreneurial intentions [28]; innovation and entrepreneurship [29]; social entrepreneurship [30]; engineering entrepreneurship education [31]; creativity, commitment, and success [26]; and creativity and growth [18]. Innovativeness at the individual level in relationship to entrepreneurial intentions [32,33], national culture [34], perceived expertise and digital marketing adoption intention of female entrepreneurs [35], and female SME performance [36] have been investigated. Other recent studies have covered various antecedents of SME growth, e.g., network relationship; financing, training, experience, and education of the manager and firm age and size [37]; human capital [38]; growth process [39]; and personality [18,24,40]. On one hand, the hypotheses in this study are novel in terms of the extant literature because they link together the three concepts in a model. On the other hand, this study upgrades earlier studies by adding growth to the curiosity–innovativeness model [41] and by adding innovativeness as a mediator in the curiosity–growth association [18]. At first glance, the curiosity–growth link may seem obvious, but this connection has not been confirmed in past research [18]. By linking the studied constructs at the level of entrepreneurs from three countries with relatively different economic dynamics and impacts on company growth, the intention is to close the gap in the literature in the field of entrepreneurial psychology and in the broader fields of entrepreneurship and psychology.

## 2. Theory and Hypotheses

Knowledge enables new products to be introduced in the market and is a decisive element in the innovation process that reduces costs, risk, and time [42]. Likar noted that knowledge and intellectual capital are becoming the most important factors in success [43]. To acquire knowledge or increase intellectual capital, a person must possess at least a certain degree of curiosity. Curiosity exposes a longing for novel information; is provoked by new, compound, or equivocal stimuli; and motivates research behavior [44]. Harvey et al. defined curiosity as the cyclical attainment of always additional information because of always additional knowledge gaps. Entrepreneurial curiosity was chosen as a construct for research because entrepreneurs need to have the necessary skills that can help them solve problems in business and ensure the path to growth [45].

Research [46,47,48] shows that innovation lies at the heart of entrepreneurship, making it a decisive element in fostering economic and social progress in emerging nations. Furthermore, in the literature, innovations can be traced that are attributed to the often-identified functional feature of entrepreneurs [3,49,50,51,52,53].

In this article, research in the field of innovation relates to the level of the entrepreneurs. Entrepreneurs are more innovative than non-entrepreneurial individuals. Agarwal and Prasad characterized personal innovation as an affection that arises in some individuals but not in others [54]. In this study, we will clearly separate the entrepreneur’s innovation from innovation at the company level to explore how the innovation of the entrepreneur is linked to the growth of their company. Entrepreneurial innovation was chosen because an entrepreneur must be able to implement ideas if they want their business to grow.

In recent times, entrepreneurial psychology has become a topical research field around the world, with considerable research (for example, [55,56]) showing that a set of entrepreneurial attributes can add to the outcomes of entrepreneurial deeds.

In this article, the first determinant of entrepreneurial behavior we will study is entrepreneurial curiosity, an encouraging emotive-motivating system aimed at research in the entrepreneurial framework, coming to grips with duties connected to entrepreneurship, and featuring novel steps that enhance a business venture [17,57]. Entrepreneurial curiosity is encouraged when an entrepreneur is challenged with diverse impetuses in the milieu connected to entrepreneurship [15].

The next determinant we will study is entrepreneurial innovation, to be considered as a dependent variable. This is an interesting construct for research that influences many results of a company. Hatak et al., for example, described the well-researched positive relationship between innovation and company performance, which applies equally to all companies [58]. Baer [59], in summarizing various authors [60,61], noted that innovation, especially in dynamic environments, is vital for the growth and competitiveness of organizations and, as such, is of interest in the inquiry on the association between entrepreneurial curiosity and innovativeness and company growth [24,62,63,64,65]. The association between entrepreneurial curiosity and innovativeness can be positive [41]. Likar described how in the manufacturing and services sectors, there is a wide and structured set of important influencing factors that lead to innovation results and thus improved business results and indicated that it is necessary to treat companies in the manufacturing and services sectors separately and to treat companies within those sectors separately based on the level of technological development or knowledge [66]. On the basis of the preceding discussion, we have operationalized Hypothesis 1:

**H1**.  *Entrepreneurial curiosity has a positive effect on the entrepreneur’s innovativeness*.

According to the above findings on innovation, it would be reasonable to conclude that company-level and entrepreneur-level innovation is essential for the growth of the company. Furthermore, this article intends to focus specifically on the study of innovation at the entrepreneur level in SMEs, given that this could raise pertinent questions about different theories of innovation.

Various authors specify innovation as the capacity, competence, and willingness of companies and their owners to build up advantages or initiate innovations or inventions in companies or in innovation projects [25,67,68]. Innovation can be generalized to the innovation of the owner or the manager, rather than innovation of the company [69]. Thakur et al. discovered that personal innovation is positively linked to technological innovation [70]. Kirzner stated that innovation is the outcome of the competence of an entrepreneur who pays attention to opportunities and exploits information asymmetries [71].

Innovation is considered one of the most essential foundations of entrepreneurship and a principal feature when describing entrepreneurs [53,72,73,74]. In this research, we will study innovation at the entrepreneur level as appraised with the Jackson Personality Inventory questionnaire [75]. Adjectives related to measuring that are used to describe entrepreneurs strongly coincide with innovation and include ingenuity, entrepreneurship, inventiveness, innovativeness, and foresight [75]. In this context, innovation is defined as identifying the attitudes or other characteristics of an individual that distinguish more innovative individuals from less innovative ones [76]. Entrepreneurial innovation refers to the characteristics of an entrepreneur as defined by the measures described above [75] and is reflected in the operation of the company, with innovation being an affirmative force for growth at the organizational level [77,78]. The creative abilities of the entrepreneur (as a foundation for innovativeness) can influence the company’s growth [18,79,80]. Innovativeness can be an important characteristic and competency of the entrepreneur. Innovativeness, initiative, and risk taking can be important competencies of SME managers, while the CEO competency is found to positively impact management performance [81]. To survive in the face of competition and sustain firm growth, the entrepreneur must build up capabilities such as innovativeness, autonomy, risk-taking orientation, and proactive orientation [82]. The individual-level innovativeness of females and SME performance tend to be related [36]. Accordingly, we have operationalized Hypothesis 2:

**H2**.  *The entrepreneur’s innovativeness has a positive effect on company growth*.

## 3. Data and Methods

We obtained data for this enquiry by sending a survey questionnaire via e-mail. The survey was processed in 2019, before the COVID-19 pandemic. The sample consisted of entrepreneurs, i.e., (co)owners or (co)founders, of enterprises with up to 250 workers. Data (e-mail addresses) on entrepreneurs suitable for the research were obtained from publicly available registers. Random sampling was used to obtain a representative set of companies in each country and their e-mail addresses (companies were randomly selected from available databases of enterprises but were limited to SMEs). The first country in which we conducted the research was Slovenia, the second was Serbia, and the third was Latvia. The lists were made of 5000 randomly selected SMEs (sorted using a new variable with a random value) from the available databases of companies in each country: the Enterprise Register of Latvia (about 181,000 SMEs), the Serbian Business Register (around 135,000 SMEs), and the Slovenian Business Register (about 74,000 SMEs). Slovenia was chosen because it is the first country in the group of post-transition countries to have enjoyed the presidency of the European Union Council and also the earliest in this set of economies to have accepted the euro as its payment currency [18]. This permits us to conclude that it has a rather strong economy and thus advanced entrepreneurship. Serbia was chosen since this country underwent a war after its conversion from a planned to a market economy and the economy is still getting back on its feet, the country has a higher unemployment rate, and it has poorer credit ratings. It is economically dissimilar to or less advanced than Slovenia, yet both countries have cultural resemblance as Slovenia and Serbia were part of the same country for decades. The third European country is Latvia, which has a different culture than the other two countries and has experienced a nonviolent conversion to a market economy. Data on entrepreneurs in a different entrepreneurially developed country (Serbia) and in a culturally different country (Latvia) were collected to help validate the models formed on the basis of the first sample. We checked the representativeness by comparing the size structure of enterprises with 250 workers or fewer in each nation and the total population of that nation.

The survey included measures of entrepreneurial curiosity ([15]; 16 questions), measures of innovation at the entrepreneur level ([75]; 8 questions), and measures of company growth, which included sales growth, growth in the number of workers, and market share growth [14,80,83,84]. The selected measures fitted well with constructs in the hypotheses. The chosen items of entrepreneurial curiosity reflected the entrepreneur’s curiosity in terms of paying attention to other entrepreneurs’ concerns and seeking information and knowledge about entrepreneurship, business, and market matters. The selected items of entrepreneur innovativeness reflected the person’s innovativeness in terms of their innovative skills, innovative thinking, and experimentation. The chosen items of enterprise growth revealed growth across three aspects (sales, employees, and market). The scales and items have been found to be valid and reliable in past research (e.g., entrepreneurial curiosity [15], innovation at the entrepreneur level [75], and growth [14,80,83,84]), which proves that the chosen measures assessed the constructs correctly. The scales used to appraise the three constructs are presented in Appendix A, including statistics of retained measurement items. The questionnaire also included control variables: gender, age, (co)ownership, (co)founding, education, industry, and company life cycle.

The data included usable responses from 851 entrepreneurs in three nations: Slovenia (n = 359), Latvia (n = 338), and Serbia (n = 154). Sample properties are shown in Appendix B. In the sample, the majority of the enterprises were small (annual sales EUR 4 million or less; up to 50 workers), mature (firm age 11 to 50 years in Slovenia and Serbia and 6 to 20 years in Latvia), in several industries (the majority in services), and predominantly in the maturity or growth phase of the company life cycle. The sample entrepreneurs were primarily female in Slovenia and Latvia and male in Serbia, meaning women were over-represented in the sample compared with the population. The majority of entrepreneurs were more than 40 years old. However, younger ones were also well represented. In Slovenia and Serbia, they mainly held a college or an undergraduate degree and in Latvia, they mainly held a graduate degree; they were owners or co-owners or founders or co-founders and predominantly in Slovenia and Latvia, while less than half were in Serbia. The samples represented the companies and entrepreneurs in the three countries well, except that they were skewed by gender toward female entrepreneurs, perhaps because females liked the questionnaire topic better than males.

The constructs were evaluated using reliability and factor analysis (exploratory and confirmatory). For statistical analysis, we used the Statistical Package for Social Sciences (SPSS) to verify the constructs and then tested the constructs and hypotheses with the help of modeling with structural equations (SEM—structural equation modeling; the EQS program). Data from the first sample (Slovenia) were used to advance the models, whereas data from the second (Serbia) and the third (Latvia) samples were used to validate the results. We responded to the research challenge with partial structural models studied on the basis of the hypotheses.

Indirect effects in the models were also assessed. Curiosity, creativity, and commitment can be considered as determinants of entrepreneurial success [26]. Entrepreneurial passion, curiosity, and innovativeness can be connected to entrepreneurial intentions [28]. Curiosity is necessary for entrepreneurs’ learning [29] and can be a determining factor of entrepreneurial success [30], but entrepreneurial curiosity and enterprise growth were not found to be directly associated [18]. On the basis of the above studies and H1 and H2, we put to test the indirect effect of entrepreneurial curiosity (through the mediation of the entrepreneur’s innovativeness) on company growth. The models were also checked with control variables by evaluating them on subsamples classified according to control variables (e.g., gender: female and male; life cycle: early and late stages; and industry: services and production).

**Figure 1:**
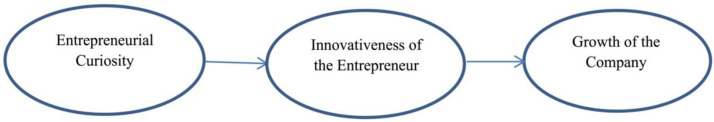
The model of entrepreneurial curiosity, innovativeness, and company growth.

Figure 1 presents the structural model considered and tested in this study and reflecting hypotheses H1 and H2 portrayed in the previous section.

## 4. Results

### 4.1. Factor Analysis and Reliability Findings

The entrepreneurial curiosity construct was put to test on the three samples (Slovenia, Latvia, and Serbia) by using factor analysis, which is exploratory in the beginning. Method: ML; rotation: Oblimin. Table 1 presents the results. Four items were retained established on basis of the magnitude of factor loadings and communalities. The Kaiser–Meyer–Olkin measure of sampling adequacy (KMO) gave adequate results (Slovenia 0.82, Latvia 0.79, and Serbia 0.76). For all three nations, the Bartlett test was significant (sig. 0.000), showing that the correlation matrix contains significant correlations. The reliability (Cronbach alpha) was good (Slovenia 0.87, Latvia 0.83, and Serbia 0.80). The exploratory factor analysis was followed by the confirmatory factor analysis (method: ERLS), which verified the findings of the exploratory factor analysis (significant, high, and positive coefficients for all items). The internal consistency of the construct was good (RHO: Slovenia 0.84, Latvia 0.82, and Serbia 0.76; Cronbach alpha reliability: Slovenia 0.87, Latvia 0.83, and Serbia 0.80) and so was the convergence, as indicated by the model goodness-of-fit indices (NFI: Slovenia 0.97, Latvia 0.98, and Serbia 0.94; CFI: Slovenia 0.98, Latvia 0.99, and Serbia 0.95; RMSEA: Slovenia 0.12, Latvia 0.09, and Serbia 0.12).

The same tests were conducted on other constructs and indicated acceptable moderate-to-good results (the innovativeness of the entrepreneur construct: Table 2, three items retained; the firm growth construct: Table A3 in Appendix A, all three items retained; Cronbach alpha: Slovenia 0.70, Latvia 0.67, and Serbia 0.70).

### 4.2. Structural Equation Modelling Findings

The postulated associations were assessed in a structural equation model (method: ERLS) on the complete data and on the three samples (Table 3 and Table 4 present the findings). The models were found to be appropriate on the whole and in all three nations:

Internal consistency

- Cronbach alpha reliability
  ○ Total 0.75
  ○ Slovenia 0.79
  ○ Latvia 0.69
  ○ Serbia 0.70
- RHO
  ○ Total 0.83
  ○ Slovenia 0.85
  ○ Latvia 0.80
  ○ Serbia 0.77

Model goodness-of-fit indices

- NFI
  ○ Total 0.98
  ○ Slovenia 0.96
  ○ Latvia 0.96
  ○ Serbia 0.89
- RMSEA
  ○ Total 0.03
  ○ Slovenia 0.05
  ○ Latvia 0.03
  ○ Serbia 0.04
- CFI
  ○ Total 0.99
  ○ Slovenia 0.98
  ○ Latvia 0.99
  ○ Serbia 0.98

Hypothesis 1 anticipated a positive link between entrepreneurial curiosity and innovativeness of the entrepreneur. Coefficients were found to be significant and positive on the whole and in all three nations (standardized coefficients: overall 0.37, Slovenia 0.46, Latvia 0.17, and Serbia 0.50). R-squared (variance explained) was found to be substantial on the whole (0.14); it was 0.25 for Serbia, 0.22 for Slovenia, and 0.03 (the worse) for Latvia. These calculations are in favor of H1.

Hypothesis 2 projected a positive association between innovativeness of the entrepreneur and firm growth. Coefficients were found to be significant and positive for the whole sample and in two of the three nations (standardized coefficients: overall 0.15, Slovenia 0.19, and Serbia 0.14). R-squared (variance explained) was discovered to be low for the whole sample (0.02), 0.03 for Slovenia, 0.02 for Serbia, and non-existent for Latvia (0.00). The findings mainly endorse H2, with the exception of Latvia.

The consistency of the structural model findings was checked by separating the whole sample by control variables (Table 5 and Table 6 display the findings). The entrepreneurial curiosity–innovativeness of the entrepreneur association coefficient was significant and positive for the sub-samples of each control variable, indicating strong support for H1. The innovativeness of the entrepreneur–growth association coefficient was positive for each sub-sample and significant for most sub-samples, showing solid support for H2.

Supplementing direct effects, some factors were uncovered in the model that led to small, indirect effects of entrepreneurial curiosity on growth through the entrepreneur’s innovativeness. Standardized coefficients for the indirect effects were positive for the total (0.06, significant), for Slovenia (0.08, significant), and for Serbia (0.07, not significant). The result was nearly null for Latvia (standardized coefficient 0.00). Therefore, the results provide mixed evidence of an indirect link between entrepreneurial curiosity (with the mediation of the entrepreneur’s innovativeness) and company growth.

## 5. Discussion

In this enquiry, a positive connection was found between entrepreneurial curiosity and innovativeness of the entrepreneur in each of the three countries. On the basis of their curiosity (e.g., being interested in other entrepreneurs’ interests, willing to know information about marketing and how a particular business system operates, and nonstop delving into entrepreneurship issues), entrepreneurs will tend to have expressed innovation interests and abilities (e.g., persons repeatedly solicit their assistance in creative works, entrepreneurs fancy work that entails original thoughts, and entrepreneurs love trying out diverse approaches to achieve the same goals). We may conclude that entrepreneurial curiosity can act as a good foundation for the innovativeness of the entrepreneur.

A positive link between innovativeness of the entrepreneur and business growth was discovered in Serbia and Slovenia, although not in Latvia. The innovativeness of the entrepreneur, expressed through innovation interests and abilities, can be a driving force for firm growth in terms of growth in the number of staff members, growth in sales, and growth in market share in selected nations (for example, in Serbia and Slovenia but not in Latvia in our research). Entrepreneurial innovativeness can be a mediator in the indirect impact of entrepreneurial curiosity on firm growth, which was discovered in one country (Slovenia) and for the total data.

This research has contributed to science the theoretically advanced and empirically verified model of entrepreneurial curiosity, innovativeness, and growth. This research constitutes a theoretic contribution and shows that the entrepreneur’s curiosity is important for their own innovativeness and that their innovativeness is important for the growth of the business. This was discovered for samples of entrepreneurs from three countries in Europe (Slovenia, Latvia, and Serbia). The one exception was the innovativeness–growth association in Latvia. The empirical findings based on the model of entrepreneurial curiosity, innovativeness of the entrepreneur, and business growth add to the normative research on enterprise growth by highlighting the importance of curiosity in predicting innovativeness at the entrepreneurial level and the significance of the entrepreneur’s innovativeness for business growth. This study also revealed some indirect significance of entrepreneurial curiosity for business growth.

The research made an empirical contribution by improving or reassessing measurement instruments of entrepreneurial curiosity [15] and innovativeness of the entrepreneur [75] in three nations and exposing the fundamental etic (comparable cross-nationally) items:

1. Entrepreneurial curiosity items (listed beneath Table 1).
2. Innovativeness of the entrepreneur items (listed beneath Table 2).

This research bears implications for research, theory, and practice. Researchers can use the two cross-nationally analogous measurement instruments in their research. The theory can concentrate more on entrepreneurial curiosity and innovativeness at the entrepreneur level for predicting firm-level growth. Practitioners and policymakers ought to understand that the characteristics of the entrepreneur related to curiosity and innovativeness can be important for the growth of companies (sales growth, growth in the number of workers, and growth in market share). Accordingly, training and education in companies and in the education system should focus increasingly on expanding the curiosity and innovativeness of persons in line to enhance business outcomes in terms of firm growth. Going by the results, entrepreneurs from the three countries are advised to advance their entrepreneurial curiosity with the purpose of improving their innovativeness. It is recommended that Slovenian and Serbian entrepreneurs increase their innovativeness so as to increase growth and Slovenian entrepreneurs foster their entrepreneurial curiosity with the aim of improving growth.

The key points are as follows: (1) The entrepreneur influences the results of their company through their actions. (2) The considered constructs of the entrepreneur were selected succeeding a review of the literature and are not the only ones that affect the entrepreneur and their operation in the company. (3) Data obtained in the survey on a sample of entrepreneurs from three countries served as a basis for statistical analysis. (4) It may be possible to transfer the results of this research to entrepreneurs in the countries in question, yet it would be more difficult to seek to apply them to entrepreneurs in other countries because of the distinctive attributes of the environment. 

The principal limitations of this research are as follows: 

(1) Number of constructs: Only a couple of psychological constructs of the entrepreneurs were taken into account and not all those that influence their behavior or business growth. This study focused on entrepreneurs’ personal characteristics and did not consider their social skills [85,86] or the fact that most entrepreneurial efforts are actually team efforts [86,87,88]. 

(2) The questionnaire: Data for the survey were attained via a closed-ended questionnaire. In the questionnaire, we used constructs already validated and used in past research, basically perceptual measures. In the curiosity and innovativeness instruments, many items were excluded and established on the magnitude of factor loadings and communalities in all three nations to ensure the comparability of constructs, yet the retained items portrayed the content of constructs well. 

(3) Data collection in the identical time period: Conclusions about causation in the hypotheses were made on the basis of the literature. With the proposed quantitative methodology, we were unable to directly verify causality because the data were gathered simultaneously with a single questionnaire, but it was possible to set structural models to verify the causal relationships given in the model hypotheses. 

(4) Applicability of the findings: The survey among entrepreneurs was conducted in three countries in Europe, meaning the findings might not be entirely applicable to all nations around the world. The context (the three countries with different economic dynamics) is important regarding the findings. All three nations belong to the group of European post-transition countries; hence, results are mainly relevant for these nations. Results were the strongest in the most advanced nation of the three, Slovenia, which is closest of the three to the Western-type economies. Therefore, the study may also have some relevance for more advanced economies. 

(5) Exclusions: The industry type was not included in sampling criteria, even if there may be a big difference in the innovativeness and growth rates of different types of firms (low-tech vs. high-tech, traditional firms vs. innovative startups, etc.) and curiosity may be less important in less innovative, less technology intensive industries. 

(6) Changes after COVID-19 lockdowns: Since the time of data collection may be relevant to the threads studied and data was gathered before the COVID-19 pandemic, this study could not address changes ascribable to the lockdowns.

We offer some proposals for future research: (1) The connections between the two individual-level constructs (entrepreneurial curiosity and innovativeness of the entrepreneur) and the firm-level growth construct could be examined more in other national contexts, by possibly supplementing selected additional personal- and firm-level variables and constructs. For example, variables of the family business transfer to the next generation can be added because they can be related to long-term success [89,90,91]. The model developed in this study and constructs in the model can be extended by adding sustainability elements [92,93,94]. (2) The cross-nationally comparable measurement instruments used in this research could be further validated and refined. (3) In-depth interviews and other qualitative research techniques may add insights about the substance and operation of the transformation of personal-level aspirations and activities (connected to curiosity and innovativeness) into firm-level effects (growth).

## 6. Conclusions

This research adds to the entrepreneurship knowledge base by delivering empirical testimony on the connections between entrepreneurial curiosity, innovativeness of the entrepreneur, and firm growth, as well as presenting polished cross-nationally analogous instruments for measuring entrepreneurial curiosity and innovativeness of the entrepreneur. The curiosity of the entrepreneur is valuable for their innovativeness. In some countries, the entrepreneur’s innovativeness is important for the company’s growth. The model developed in this research could be refined and extended in future investigations.

## Figures and Tables

**Table 1:** The entrepreneurial curiosity construct—factor analysis and reliability results.

Factor Analysis *		Sample		
Exploratory (method: ML; rotation: Oblimin)		Slovenia (n = 359)	Serbia (n = 154)	Latvia (n = 338)
KMO		0.82	0.76	0.79
Bartlett test	Chi square	665.04	186.92	519.91
	df	6	6	6
	Sig.	0.000	0.000	0.000
Total variance explained		61.94%	50.25%	56.26%
Reliability	Cronbach alpha	0.87	0.80	0.83
Confirmatory (method: ERLS)				
model	Chi square	17.39	9.94	10.97
	df	3	3	3
	Sig.	0.001	0.019	0.012
Goodness-of-fit	NFI	0.97	0.94	0.98
	RMSEA	0.12	0.12	0.09
	CFI	0.98	0.95	0.99
Reliability	Cronbach alpha	0.87	0.80	0.83
	RHO	0.84	0.76	0.82

* Items (Agreement with the statement: 1 = Strongly Disagree, 2 = Moderately Disagree, 3 = Slightly Disagree, 4 = Neither Agree Nor Disagree, 5 = Slightly Agree, 6 = Moderately Agree, 7 = Strongly Agree): I continuously delve into entrepreneurship matters; I simply must know how a certain business system works; In my business, I must have information about marketing that is as complete as possible; I am interested in other entrepreneurs’ interests.

**Table 2:** The innovativeness of the entrepreneur construct—factor analysis and reliability results.

Factor Analysis *		Sample		
Exploratory (method: ML; rotation: Oblimin)		Slovenia (n = 359)	Serbia (n = 154)	Latvia (n = 338)
KMO		0.67	0.61	0.60
Bartlett test	Chi square	287.78	38.75	174.14
	df	3	3	3
	Sig.	0.000	0.000	0.000
Total variance explained		53.78%	31.68%	46.13%
Reliability	Cronbach alpha	0.76	0.56	0.67
Confirmatory (method: ERLS)				
model	Chi square	0.01	1.93	4.04
	df	1	1	1
	Sig.	0.907	0.164	0.044
Goodness-of-fit	NFI	1.00	0.93	0.98
	RMSEA	0.00	0.08	0.09
	CFI	1.00	0.96	0.98
Reliability	Cronbach alpha	0.76	0.56	0.66
	RHO	0.77	0.61	0.71

* Items (Agreement with the statement: 1 = Strongly Disagree, 2 = Moderately Disagree, 3 = Slightly Disagree, 4 = Neither Agree Nor Disagree, 5 = Slightly Agree, 6 = Moderately Agree, 7 = Strongly Agree): I like to experiment with various ways of doing the same thing; I prefer work that requires original thinking; People often ask me for help in creative activities.

**Table 3:** Structural equation modeling results (standardized coefficients and variance explained).

Sample (n)	EC–INN	INN–GR	R2INN	R2GR
Total (851)	0.37 *	0.15 *	0.14	0.02
Slovenia (359)	0.46 *	0.19 *	0.22	0.03
Serbia (154)	0.50 *	0.14 *	0.25	0.02
Latvia (338)	0.17 *	−0.01	0.03	0.00

* *p* < 0.05 (two-sided); EC–INN: entrepreneurial curiosity–innovativeness of the entrepreneur relationship coefficient; INN–GR: innovativeness of the entrepreneur–growth relationship coefficient; R2INN: variance explained (R-squared) of innovativeness of the entrepreneur; R2GR: variance explained (R-squared) of firm growth.

**Table 4:** Structural equation modeling results (goodness-of-fit and reliability).

Sample (n)	Chi	df	Sig.	NFI	RMSEA	CFI	RHO	Cronbach Alpha
Total (851)	57.48	34	0.007	0.98	0.03	0.99	0.83	0.75
Slovenia (359)	59.27	34	0.005	0.96	0.05	0.98	0.85	0.79
Serbia (154)	40.64	34	0.201	0.89	0.04	0.98	0.77	0.70
Latvia (338)	42.07	34	0.161	0.96	0.03	0.99	0.80	0.69

**Table 5:** Structural equation modeling results—controls (coefficients and variance explained).

Control Group	(n)	EC–INN	INN–GR	R2INN	R2GR
Gender	Male (387)	0.33 *	0.24 *	0.11	0.06
	Female (464)	0.40 *	0.07	0.16	0.01
Age	Younger: over 20–50 years (507)	0.29 *	0.16 *	0.08	0.03
	Older: over 50 years (344)	0.50 *	0.14 ·	0.25	0.02
Education	Up to undergraduate (599)	0.43 *	0.13 *	0.18	0.02
	Graduate degree (252)	0.22 *	0.20 *	0.05	0.04
Founder or co-founder	Yes (564)	0.43 *	0.16 *	0.19	0.02
	No (287)	0.24 *	0.19 *	0.06	0.03
Owner or co-owner	Yes (640)	0.39 *	0.14 *	0.15	0.02
	No (211)	0.31 *	0.22 *	0.10	0.05
Industry	Manufacturing (149)	0.49 *	0.31 *	0.24	0.10
	Services (702)	0.35 *	0.11 *	0.12	0.01
Firm age	0–10 years (329)	0.30 *	0.13 ·	0.09	0.02
	11 or more years (522)	0.41 *	0.16 *	0.17	0.02
Size	0–10 employees (631)	0.29 *	0.10 ·	0.08	0.01
	11–250 employees (220)	0.57 *	0.09	0.32	0.01
Stage in the life cycle	Startup–growth (312)	0.28 *	0.17 *	0.08	0.03
	Maturity and later (539)	0.42 *	0.19 *	0.17	0.01

* *p* < 0.05 (two-sided), ·*p* < 0.10 (two-sided); EC–INN: entrepreneurial curiosity–innovativeness of the entrepreneur relationship coefficient; INN–GR: innovativeness of the entrepreneur–growth relationship coefficient; R2INN: variance explained (R-squared) of innovativeness of the entrepreneur; R2GR: variance explained (R-squared) of firm growth.

**Table 6:** Structural equation modeling results—controls (goodness-of-fit and reliability).

Control Group	(n)	Chi	df	Sig.	NFI	RMSEA	CFI	RHO	Cronbach Alpha
Gender	Male (387)	27.76	34	0.766	0.98	0.00	1.00	0.83	0.75
	Female (464)	55.74	34	0.011	0.96	0.04	0.98	0.82	0.74
Age	Younger: over 20–50 years (507)	73.42	34	0.000	0.96	0.05	0.98	0.82	0.74
	Older: over 50 years (344)	21.82	34	0.947	0.98	0.00	1.00	0.84	0.76
Education	Up to undergraduate (599)	64.27	34	0.001	0.97	0.04	0.98	0.83	0.75
	Graduate degree (252)	36.52	34	0.352	0.95	0.02	1.00	0.82	0.73
Founder or co-founder	Yes (564)	45.76	34	0.086	0.98	0.02	0.99	0.84	0.77
	No (287)	40.62	34	0.202	0.95	0.03	0.99	0.81	0.71
Owner or co-owner	Yes (640)	37.31	34	0.319	0.98	0.01	1.00	0.83	0.76
	No (211)	42.04	34	0.162	0.93	0.03	0.99	0.81	0.72
Industry	Manufacturing (149)	27.24	34	0.787	0.94	0.00	1.00	0.85	0.77
	Services (702)	61.13	34	0.003	0.97	0.03	0.99	0.82	0.74
Firm age	0–10 years (329)	35.61	34	0.393	0.97	0.01	1.00	0.83	0.75
	11 or more years (522)	43.43	34	0.129	0.97	0.02	0.99	0.82	0.75
Size	0–10 employees (631)	48.31	34	0.053	0.97	0.03	0.99	0.82	0.73
	11–250 employees (220)	47.31	34	0.064	0.93	0.04	0.98	0.82	0.73
Stage in the life cycle	Startup–growth (312)	36.35	34	0.360	0.96	0.01	1.00	0.81	0.73
	Maturity and later (539)	48.32	34	0.053	0.97	0.03	0.99	0.83	0.76

## Data Availability

Data are available on request from the authors.

## Appendix A. Measurement Items

**Table A1:** Entrepreneurial curiosity items* (Agreement with the statement: 1 = Strongly Disagree, 2 = Moderately Disagree, 3 = Slightly Disagree, 4 = Neither Agree nor Disagree, 5 = Slightly Agree, 6 = Moderately Agree, 7 = Strongly Agree) [15].

Item	Country	Mean	Standard Deviation
I am interested in other entrepreneurs’ interests.	Slovenia	4.79	1.61
	Serbia	5.14	1.80
	Latvia	4.78	1.70
In my business, I must have information about marketing that is as complete as possible.	Slovenia	5.08	1.65
	Serbia	5.32	1.79
	Latvia	5.04	1.71
I simply must know how a certain business system works.	Slovenia	5.02	1.66
	Serbia	5.64	1.45
	Latvia	5.13	1.78
I continuously delve into entrepreneurship matters.	Slovenia	4.75	1.71
	Serbia	5.01	1.68
	Latvia	4.76	1.63

* Excluded items: I explore new things that could create additional profit. In entrepreneurial work, I am mostly interested in competition. I am very interested in knowing the needs I can meet in society. I am able to create added value from my observations of the environment. I spend most of my time thinking about company improvements. Frequency of occurrence: 1 = Never, 2 = Very Rarely, 3 = Rarely, 4 = Occasionally, 5 = Often, 6 = Very Often, 7 = Always. I enjoy conversations about obtaining capital for the firm. It bores me to always watch the same products; therefore, I think about improving and offering them to the market. When I notice an abandoned building, I think about what business potential it represents for me. While doing market research, I focus on the work so much that I lose track of time. I spend hours on a problem because I cannot rest without an answer. Conceptual problems keep me awake thinking about solutions. When I have some free time, I spend it researching new markets.

**Table A2:** Innovativeness of the entrepreneurial items* (Agreement with the statement: 1 = Strongly Disagree, 2 = Moderately Disagree, 3 = Slightly Disagree, 4 = Neither Agree Nor Disagree, 5 = Slightly Agree, 6 = Moderately Agree, 7 = Strongly Agree) [75].

Item	Country	Mean	Standard Deviation
People often ask me for help in creative activities.	Slovenia	4.98	1.49
	Serbia	5.64	1.57
	Latvia	4.90	1.44
I prefer work that requires original thinking.	Slovenia	5.41	1.41
	Serbia	5.79	1.49
	Latvia	5.23	1.42
I like to experiment with various ways of doing the same thing.	Slovenia	4.82	1.48
	Serbia	5.40	1.63
	Latvia	4.46	1.69

* Excluded items: I like a job that demands skill and practice rather than inventiveness. (r). I usually continue doing a new job in exactly the way it was taught to me. (r). I obtain more satisfaction from mastering a skill than coming up with a new idea. (r). I often surprise people with my novel ideas. I do not really think of myself as a creative person. (r).

**Table A3:** Growth items [14,80,83,84].

Item	Country	Mean	Standard Deviation
Average annual growth in sales over the last three years (1 = less than 5%, 2 = 5%–9%, 3 = 10%–19%, 4 = 20%–34%, 5 = 35%–50%, 6 = more than 50%).	Slovenia	2.43	1.32
	Serbia	2.74	1.33
	Latvia	1.91	1.05
Average annual growth in the number of employees over the last three years (1 = less than 0%, 2 = 0%–4%, 3 = 5%–9%, 4 = 10%–19%, 5 = 20%–35%, 6 = more than 35%).	Slovenia	2.18	1.29
	Serbia	2.38	1.40
	Latvia	1.72	0.90
Growth in market share over the last three years (the market share of your company is ... 1 = decreasing, 2 = holding its own, 3 = increasing slightly, 4 = increasing moderately, 5 = increasing significantly).	Slovenia	2.60	0.92
	Serbia	3.00	1.21
	Latvia	2.49	1.06

## Appendix B. Sample Characteristics

**Table A4:** Number of employees (full-time equivalent).

Country	Group	Frequency	Percent	Percent in the Population [95,96,97]
Slovenia	0–50	346	96.4	99.2
	51–250	13	3.6	0.8
	Total	359	100.0	100.0
Serbia	0–50	150	97.4	99.3
	51–250	4	2.6	0.7
	Total	154	100.0	100.0
Latvia	0–50	331	97.9	98.7
	51–250	7	2.1	1.3
	Total	338	100.0	100.0
All	0–50	827	97.2	/
	51–250	24	2.8	/
	Total	851	100.0	/

**Table A5:** Total sales in the last finished calendar year.

Country	Group	Frequency	Percent
Slovenia	EUR 400,000 or less	214	59.6
	Over EUR 400,000–EUR 800,000	46	12.8
	Over EUR 800,000–EUR 1,600,000	36	10.0
	Over EUR 1,600,000–EUR 4,000,000	33	9.2
	Over EUR 4,000,000–EUR 20,000,000	25	7.0
	Over EUR 20,000,000	5	1.4
	Total	359	100.0
Serbia	Over EUR 400,000–EUR 800,000	105	68.2
	Over EUR 800,000–EUR 1,600,000	27	17.5
	Over EUR 1,600,000–EUR 4,000,000	18	11.7
	Over EUR 4,000,000–EUR 20,000,000	4	2.6
	Total	154	100.0
Latvia	EUR 400,000 or less	275	81.4
	Over EUR 400,000–EUR 800,000	27	8.0
	Over EUR 800,000–EUR 1,600,000	12	3.6
	Over EUR 1,600,000–EUR 4,000,000	12	3.6
	Over EUR 4,000,000–EUR 20,000,000	9	2.7
	Over EUR 20,000,000	3	0.9
	Total	338	100.0
All	EUR 400,000 or less	489	57.5
	Over EUR 400,000–800,000 EUR	178	20.9
	Over EUR 800,000–EUR 1,600,000	75	8.8
	Over EUR 1,600,000–EUR 4,000,000	63	7.4
	Over EUR 4,000,000–EUR 20,000,000	38	4.5
	Over EUR 20,000,000	8	0.9
	Total	851	100.0

**Table A6:** Age of the company (in years).

Country	Group	Frequency	Percent
Slovenia	0–1	4	1.1
	2–5	41	11.4
	6–10	83	23.1
	11–20	100	27.9
	21–50	116	32.3
	More than 50	15	4.2
	Total	359	100.0
Serbia	0–1	3	1.9
	2–5	5	3.2
	6–10	20	13.0
	11–20	62	40.3
	21–50	54	35.1
	More than 50	10	6.5
	Total	154	100.0
Latvia	0–1	23	6.8
	2–5	59	17.5
	6–10	91	26.9
	11–20	111	32.8
	21–50	54	16.0
	Total	338	100.0
All	0–1	30	3.5
	2–5	105	12.3
	6–10	194	22.8
	11–20	273	32.1
	21–50	224	26.3
	More than 50	25	2.9
	Total	851	100.0

**Table A7:** Type of business.

Country	Group	Frequency	Percent
Slovenia	Banking, investment, insurance	7	1.9
	Construction	24	6.7
	Consumer services	89	24.8
	Manufacturing consumer goods	40	11.1
	Manufacturing industrial goods	33	9.2
	Engineering, research and development	17	4.7
	Mining, extraction, oil	18	5.0
	Management consulting and business services	48	13.4
	Retail or wholesale trade	19	5.3
	Transportation or public utilities	64	17.8
	Total	359	100.0
Serbia	Banking, investment, insurance	4	2.6
	Construction	13	8.4
	Consumer services	34	22.1
	Manufacturing consumer goods	14	9.1
	Manufacturing industrial goods	16	10.4
	Engineering, research and development	10	6.5
	Mining, extraction, oil	1	0.6
	Management consulting and business services	35	22.7
	Retail or wholesale trade	12	7.8
	Transportation or public utilities	15	9.7
	Total	154	100.0
Latvia	Banking, investment, insurance	1	0.3
	Construction	38	11.2
	Consumer services	108	32.0
	Manufacturing consumer goods	25	7.4
	Manufacturing industrial goods	21	6.2
	Engineering, research and development	11	3.3
	Mining, extraction, oil	3	0.9
	Management consulting and business services	50	14.8
	Retail or wholesale trade	45	13.3
	Transportation or public utilities	36	10.7
	Total	338	100.0
All	Banking, investment, insurance	12	1.4
	Construction	75	8.8
	Consumer services	231	27.1
	Manufacturing consumer goods	79	9.3
	Manufacturing industrial goods	70	8.2
	Engineering, research and development	38	4.5
	Mining, extraction, oil	22	2.6
	Management consulting and business services	133	15.6
	Retail or wholesale trade	76	8.9
	Transportation or public utilities	115	13.5
	Total	851	100.0

**Table A8:** Stage in the life cycle of the company.

Country	Group	Frequency	Percent
Slovenia	Startup	7	1.9
	Growth	131	36.5
	Maturity	148	41.2
	Renewal	51	14.2
	Decline	21	5.8
	Failure	1	0.3
	Total	359	100.0
Serbia	Startup	3	1.9
	Growth	53	34.4
	Maturity	76	49.4
	Renewal	16	10.4
	Decline	6	3.9
	Total	154	100.0
Latvia	Startup	39	11.5
	Growth	79	23.4
	Maturity	183	54.1
	Renewal	27	8.0
	Decline	10	3.0
	Total	338	100.0
All	Startup	49	5.8
	Growth	263	30.9
	Maturity	407	47.8
	Renewal	94	11.0
	Decline	37	4.3
	Failure	1	0.1
	Total	851	100.0

**Table A9:** Gender.

Country	Group	Frequency	Percent
Slovenia	Male	131	36.5
	Female	228	63.5
	Total	359	100.0
Serbia	Male	90	58.4
	Female	64	41.6
	Total	154	100.0
Latvia	Male	166	49.1
	Female	172	50.9
	Total	338	100.0
All	Male	387	45.5
	Female	464	54.5
	Total	851	100.0

**Table A10:** Age of the person (in years).

Country	Group	Frequency	Percent
Slovenia	Over 20–30	5	1.4
	Over 30–40	83	23.1
	Over 40–50	125	34.8
	Over 50	146	40.7
	Total	359	100.0
Serbia	Over 20–30	19	12.3
	Over 30–40	43	27.9
	Over 40–50	47	30.5
	Over 50	45	29.2
	Total	154	100.0
Latvia	Over 20–30	20	5.9
	Over 30–40	71	21.0
	Over 40–50	94	27.8
	Over 50	153	45.3
	Total	338	100.0
All	Over 20–30	44	5.2
	Over 30–40	197	23.1
	Over 40–50	266	31.3
	Over 50	344	40.4
	Total	851	100.0

**Table A11:** Achieved educational level.

Country	Group	Frequency	Percent
Slovenia	Primary or high school	84	23.4
	College, undergraduate degree	231	64.3
	Graduate degree	44	12.3
	Total	359	100.0
Serbia	Primary or high school	44	28.6
	College, undergraduate degree	91	59.1
	Graduate degree	19	12.3
	Total	154	100.0
Latvia	Primary or high school	32	9.5
	College, undergraduate degree	117	34.6
	Graduate degree	189	55.9
	Total	338	100.0
All	Primary or high school	160	18.8
	College, undergraduate degree	439	51.6
	Graduate degree	252	29.6
	Total	851	100.0

**Table A12:** Founder or co-founder of the company.

Country	Group	Frequency	Percent
Slovenia	Yes	298	83.0
	No	61	17.0
	Total	359	100.0
Serbia	Yes	70	45.5
	No	84	54.5
	Total	154	100.0
Latvia	Yes	196	58.0
	No	142	42.0
	Total	338	100.0
All	Yes	564	66.3
	No	287	33.7
	Total	851	100.0

**Table A13:** Owner or co-owner of the company.

Country	Group	Frequency	Percent
Slovenia	Yes	298	83.0
	No	61	17.0
	Total	359	100.0
Serbia	Yes	73	47.4
	No	81	52.6
	Total	154	100.0
Latvia	Yes	269	79.6
	No	69	20.4
	Total	338	100.0
All	Yes	640	75.2
	No	211	24.8
	Total	851	100.0
